# Charlotte Mew: melancholy poet

**DOI:** 10.1192/bjb.2022.75

**Published:** 2023-10

**Authors:** John Callender

**Affiliations:** University of Aberdeen, UK

**Keywords:** Charlotte Mew, poetry, mental illness, psychosis, suicide

## Abstract

The main purpose of this article is to draw the attention of its readers to Charlotte Mew, a poet who is not well-known but whose work should be of great interest to mental health clinicians. She lived most of her life in the shadow of mental illness. Her poetry provides penetrating insights into her experiences of this in herself and in members of her family.

Charlotte Mew is a poet who is not widely read but whose work has been the subject of revived interest in recent years.^1^ A new biography, *This Rare Spirit: A Life of Charlotte Mew*, by Julia Copus was published in 2021.^2^ She was the subject of a *London Review of Books* podcast in 2022.^3^ Charlotte Mew wrote poetry of striking originality and beauty. To read her work is to engage with a sensibility that is unique, powerful and unforgettable.

She was born in 1869, the third eldest of seven siblings. The Mew family came from the Isle of Wight but Charlotte spent her life in London. One brother died before she was born. In March of 1876, another brother died aged 4 months. In December of the same year, a third brother died at the age of 5. After the death of her father in 1898, she lived with her mother and sister, Anne, until each in turn died.

Her mother was a demanding, dependent woman. They were perennially short of money and struggled to maintain the level of respectability that her mother expected. Anne was a talented painter, who had trained at the Royal Female School of Art, London. Rather than devoting herself to creative work, she had to spend much of her life in paid employment that involved tasks such as restoring painted furniture and decorating mirrors.

There is no record of Charlotte having any romantic attachments. She appears to have become mentally ill in the final months of her life and died by suicide.

## Lyricism and synaesthesia

Her poetry is pervaded by themes of loss, loneliness, unrequited passion and yearning for oblivion. There are also passages of lyrical beauty, such as the poem ‘In the Fields’:

- Lord, when I look at lovely things which pass,
  - Under old trees the shadow of young leaves
- Dancing to please the wind along the grass,
  - Or the golden stillness of the August sun on the August sheaves;
- Can I believe there is a heavenlier world than this?
  - And if there is
- Will the strange heart of any everlasting thing
  - Bring me these dreams that take my breath away?
- They come at evening with the home-flying rooks and the scent of hay,
  - Over the fields. They come in Spring.

In some poems, the boundaries of the self seem to dissolve and the poet becomes part of what she is describing. The poem ‘The Changeling’ includes the passage:

- Because in the long, still dusks of Spring
- You can hear the whole world whispering;
  - The shy green grasses making love,
  - The feathers grow on the dear, grey dove,
  - The tiny heart of the redstart beat,
  - The patter of the squirrel's feet,
- The pebbles pushing in the silver streams,
- The rushes talking in their dreams [ … ]

At times, this sensitivity becomes synaesthetic. The following lines appear in the poem ‘Madeleine in Church’:

- I could hardly bear
- The dreams upon the eyes of white geraniums in dusk
  - The thick, close voice of musk,
  - The jessamine music on the thin night air [ … ]
- To know how jewels taste, just as I used to think
- There was the scent in every red and yellow rose
  - Of all the sunsets

## The shadow of mental illness

Charlotte's life was intimately intertwined with mental illness. A brother and sister suffered from severe, chronic mental illnesses. Her father, who was an architect, was prone to episodes of ‘black depression’ throughout his life, and withdrew from professional practice in his later years. He died of stomach cancer at the age of 65.

Charlotte Mew’s older brother Henry and younger sister Freda both became mentally ill in early adult life. Henry exhibited bizarre behaviour such as open and compulsive masturbation, and delusional thinking. This continued at home for 9 months until June 1884, when he was admitted to the Bethlem Hospital, at age 19. The clinical picture was one that would now be diagnosed as severe schizophrenia. He was discharged in July 1885. The final entry in his medical record states that he was ‘uncured’^2^ (p. 72). The following month, he was admitted to the Holloway Sanatorium. He remained there until 1898, when he was transferred to the Peckham House asylum, where he died of tuberculosis 3 years later.

Freda was Charlotte's youngest sister. Prior to becoming unwell, she was said to have been ‘remarkable’ and ‘like a flame’. She was described as the loveliest of the three Mew sisters and possessed of a ‘calm, disarming gaze, vibrant personality and abundant chestnut-coloured hair’^2^ (p. 229).

Freda showed a similar clinical picture to that of her brother. She also became unwell at the age of 19 and suffered from delusions and hallucinations. In February 1899, she attempted suicide and was admitted to what was then called the Isle of Wight (County) Lunatic Asylum. In the first days and weeks after admission, she would spend long hours with a book open on her lap, gazing at it without reading a word. She was apathetic and socially withdrawn but also had periods of agitation. She would sometimes jump out of bed to get at the windows. She would seize articles of clothing worn by nurses and struggle to take them, saying they belonged to her. By August 1900, she was spending most of her days sitting in the same spot, never moving, noticing anyone or saying anything. By November of the following year, she was even worse and was described as ‘perfectly stuporous’ and ‘dribbles, unable to attend to her personal wants’^2^ (p. 134). She never improved and remained in hospital until the end of her life, 60 years later.

## ‘The Farmer's Bride’

Charlotte Mew's most famous poem, and the one that brought her to prominence, was ‘The Farmer's Bride’. The narrator is a young farmer who describes all that has changed in his bride since the day they were married:

- Three Summers since I chose a maid,
- Too young maybe – but more's to do
- At harvest time than bide and woo.
  - When us was wed she turned afraid
- Of love and me and all things human;
- Like the shut of a winter's day
- Her smile went out, and ’twasn't a woman –
  - More like a frightened little fay.
    - One night in the Fall, she runned away.
- ‘Out ’mong the sheep, her be,’ they said
- ’Should properly have been abed;
- But sure enough she wasn't there
- Lying awake with her wide brown stare.
- So over seven-acre field and up-along across the down
- We chased her, flying like a hare
- Before our lanterns. To Church-Town
  - All in a shiver and a scare
- We caught her, fetched her home at last
  - And turned the key upon her, fast.
- She does the work about the house
- As well as most, but like a mouse:
  - Happy enough to chat and play
  - With birds and rabbits and such as they,
  - So long as men-folk keep away.
- ‘Not near, not near!’ her eyes beseech
- When one of us comes within reach,
  - The women say that beasts in stall
  - Look round like children at her call.
  - *I've* hardly heard her speak at all.
- Shy as a leveret, swift as he,
- Straight and slight as a young larch tree,
- Sweet as the first wild violets, she,
- To her wild self. But what to me?
- The short days shorten and the oaks are brown,
  - The blue smoke rises to the low grey sky,
- One leaf in the still air falls slowly down,
  - A magpie's spotted feathers lie
- On the black earth spread white with rime,
- The berries redden up to Christmas-time.
  - What's Christmas-time without there be
  - Some other in the house than we!
  - She sleeps up in the attic there
  - Alone, poor maid. ’Tis but a stair
- Betwixt us. Oh! my God! the down,
- The soft young down of her, the brown,
- The brown of her – her eyes, her hair, her hair!

The standard interpretation of this poem is that it is about gender, misogyny and power.^4^ Charlotte Mew's two biographers, Penelope Fitzgerald^5^ (p. 105) and Julia Copus^2^ (pp. 104, 191) propose that the farmer's bride is recoiling from the prospect of sexual intimacy and that she is, or will become, a victim of domestic abuse. The former interprets the final verse as expressing the farmer's sexual frustration and proposes this is a precursor to marital rape. One review states that the unasked question in the poem is how long the farmer ‘will be able to resist raping her?’.^6^

There is much in the poem that goes against this interpretation. The tone of the monologue is tender and wistful. The poem is imbued with a sense of painful loss. The farmer cannot understand what has happened to his wife and has no idea how he can be of help. He seems captivated by the beauty and nature of his bride (‘Sweet as the first wild violets, she,/to her wild self.’)

Abusive male partners do not think or feel in these ways. They are domineering bullies, who maintain their dominance by means such as sarcasm, violence (or the threat of it), coercion, and disparagement and humiliation of their partners.

It is unlikely that a country girl would go into marriage with no knowledge of sexuality and that she would react so badly when confronted by this. There is nothing that indicates that the farmer has mistreated his wife in any way. That she is still a ‘bride’ rather than a wife, and is described as a ‘poor maid’ in the final stanza, indicate that the marriage is unconsummated, even though it is three summers since they were married.

My reaction on first reading the poem was that it describes a young woman who has become mentally ill. She grew afraid not only of her husband but of ‘all things human’. There is an abrupt change in her demeanour (‘Like the shut of a winter's day/Her smile went out and ’twasn't a woman – /More like a little frightened fay’).

The description of her running away after dark, with no provocation and no plan about where to go, is more indicative of mental illness than an abused wife making her escape. The fact that she was brought back, not just by the farmer but by unnamed others, presumably neighbours or friends, and locked up also points in this direction. It is sometimes the case that abused partners are physically confined but this is unusual. It is even more unlikely that it would happen with the connivance of the neighbourhood.

In contrast, family members who became mentally ill at that time would sometimes be confined at home, as the only alternative was admission to an asylum. Before being admitted to hospital, Charlotte's brother Henry had at times been locked in his bedroom. In one of the most famous 19th-century novels, *Jane Eyre,* Mrs Rochester is mentally ill and spends 10 years locked in a room in the home of her husband.

I would propose that the subject of this poem is Charlotte's sister Freda. In her poetry, Charlotte Mew would sometimes draw on events from her life but would disguise this by transpositions of context and character. One of her best-known poems is ‘In Nunhead Cemetery’. The narrator is young man who is attending the burial of his sweetheart. His emotions are described with an intensity that approaches derangement. The likely source of this is the burial of Charlotte's brother Henry, which had taken place in Nunhead Cemetery 12 years before.

After the onset of her illness, Freda became socially withdrawn and exhibited bouts of agitation. One striking physical feature was her chestnut-brown hair, and the brown hair of the farmer's bride is the focus of intense attention in the final two lines of the poem. Her ‘wide brown stare’ may be a recollection of Freda sitting gazing at a book without reading it. Freda turned away from all things human. She rarely spoke (‘*I’ve* hardly heard her speak at all’). In her case record, there are descriptions of Freda running towards windows, presumably with the intention of making an escape from the hospital. There may have been times when she did succeed in running away and would have been forcibly returned.

## Stigma and eugenics

The poem ‘Ken’ describes someone with what seems to be intellectual disability, who is taken to an institution. Julia Copus points out that Freda's middle name was Kendall and suggests that this poem drew its inspiration from Charlotte Mew's experiences with her sister^2^ (p. 218). The final lines may express her feelings at walking away and leaving her sister in hospital.

- So, when they took
  - Ken to that place, I did not look
- After he called and turned on me
- His eyes. These I shall see –

The mental illnesses of family members were often closely guarded secrets because of the stigma that attached to them, and this was the case with the Mew family. The poem ‘On the Asylum Road’ describes meeting a group of psychiatric patients walking outside a hospital:

- Theirs is the house whose windows – every pane –
  - Are made of darkly stained or clouded glass:
- Sometimes you come upon them in the lane,
- The saddest crowd that you will ever pass.
- But still we merry town or village folk
  - Throw to their scattered stare a kindly grin,
- And think no shame to stop and crack a joke
  - With the incarnate wages of man's sin.

Although the tone is sympathetic, the possibility that mental illness may be a result of moral degeneracy is expressed clearly in the final line of this quotation.

Another prominent idea at that time was eugenics. It was widely believed that mental disorders were caused by heredity. Charlotte had friends in common with Marie Stopes, the feminist and pioneer of birth control. Stopes had views on eugenics that would be regarded as extreme and unacceptable today. In her book *Radiant Motherhood: A Book for Those Who are Creating the Future,* published in 1920, Stopes writes about the need to curtail the fertility of those whose ‘offspring must be physically and mentally tainted’^2^ (p. 158).

Both Charlotte and her sister Anne decided, in the light of what had happened to their brother and sister, that they should not have children, to avoid passing the risk of mental illness to the next generation. The farmer's declamation ‘What's Christmas-time without there be/Some other in the house than we!’ may be an expression of the anguish caused by Charlotte Mew's voluntary childlessness.

## Death as an escape

Charlotte Mew's poems contain many references to death. This is often expressed as a yearning for peace and calm. The poem ‘Not for that City’ ends with the lines:

- And if for anything we greatly long,
  - It is for some remote and quiet stair
- Which winds to silence and a space of sleep
- Too sound for waking and for dreams too deep.

In ‘Smile, Death’, she symbolises death as a fellow skater:

- On, on let us skate past the sleeping willows dusted with snow;
- Fast, fast down the frozen stream, with the moor and the road and the vision behind,
  - (Show me your face, why the eyes are kind!)
- And we will not speak of life or believe in it or remember it as we go.

In the light of Charlotte Mew's troubled life, it is hardly surprising that she would view death as an escape to a place of peace, or a benign companion. In the event, her death was not a kind one.

She became depressed in mood following the death from cancer of her sister Anne in June 1927. She made a will that included the instruction that a main artery was to be severed, to confirm that she was dead, before she was placed in her coffin. She tormented herself because she had not done the same thing to her sister, who might, in consequence, have been buried alive. (Although unusual, this was sometimes done at that time.) She began to worry that her sister had not died of cancer but had been infected by little black specks that she could see in her sister's studio. She worried that she also would be contaminated by these specks. She was sleeping poorly, and began to neglect herself and to lose weight.

Her doctor sent her to see a specialist to assess whether she could be certified and admitted to a mental hospital. He decided that she could not. She was therefore admitted voluntarily to a nursing home and was placed in a dismal room whose only window looked on to a blank wall.

On 24 March 1928, she went out and bought a bottle of Lysol, a corrosive disinfectant. When she returned to her room, she poured half the bottle into a glass, consumed it and lay down on her bed. She was visited by her doctor, who found her in terrible pain. Her last words to him were ‘Don't keep me, let me go’. A subsequent inquest recorded a verdict of suicide while of unsound mind.

## Data Availability

Data availability is not applicable to this article as no new data were created or analysed in this study.

## About the author

**John Callender** is a ‘working retired’ consultant psychiatrist at the Royal Cornhill Hospital, Aberdeen, and an honorary senior lecturer in the School of Medicine, Medical Sciences and Nutrition at the University of Aberdeen, UK.
